# Enriching Urea with Nitrogen Inhibitors Improves Growth, N Uptake and Seed Yield in Quinoa (*Chenopodium quinoa* Willd) Affecting Photochemical Efficiency and Nitrate Reductase Activity

**DOI:** 10.3390/plants11030371

**Published:** 2022-01-29

**Authors:** Hafeez ur Rehman, Hesham F. Alharby, Hassan S. Al-Zahrani, Atif A. Bamagoos, Nadiah B. Alsulami, Nadiyah M. Alabdallah, Tahir Iqbal, Abdul Wakeel

**Affiliations:** 1 Department of Agronomy, University of Agriculture, Faisalabad 38040, Pakistan; tahiriqbaldgk29@gmail.com; 2 Department of Biological Sciences, Faculty of Science, King Abdulaziz University, Jeddah 21589, Saudi Arabia; halharby@kau.edu.sa (H.F.A.); hsalzahrani@kau.edu.sa (H.S.A.-Z.); abamagoos@kau.edu.sa (A.A.B.); nbalsulami@kau.edu.sa (N.B.A.); 3 Department of Biology, College of Science, Imam Abdulrahman Bin Fasial University, P.O. Box 1982, Dammam 31441, Saudi Arabia; nmalabdallah@iau.edu.sa; 4 Department of Botany, University of Agriculture, Faisalabad 38040, Pakistan; 5 Institute of Soil and Environmental Sciences, University of Agriculture, Faisalabad 38040, Pakistan; abdulwakeel77@gmail.com

**Keywords:** nitrogen use efficiency, inhibitors, chlorophyll fluorescence, grain protein

## Abstract

Quinoa is a climate resilience potential crop for food security due to high nutritive value. However, crop variable response to nitrogen (N) use efficiency may lead to affect grain quality and yield. This study compared the performance of contrasting quinoa genotypes (UAF Q-7, EMS-line and JQH1) to fertilizer urea enriched with urease and nitrification inhibitors (NIs; 1% (*w*/*w*) thiourea + boric acid + sodium thiosulphate), ordinary urea and with no N as control. Application of NIs-enriched urea improved plant growth, N uptake and chlorophyll values in quinoa genotype UAF-Q7 and JHQ1, however, highest nitrate reductase (NR) activity was observed in EMS-line. Quinoa plants supplied with NIs-enriched urea also completed true and multiple leaf stage, bud formation, flowering, and maturity stages earlier than ordinary urea and control, nevertheless, all quinoa genotypes reached true and multiple leaf stage, flowering and maturity stages at same time. Among photosynthetic efficiency traits, application of NIs-enriched urea expressed highest photosynthetic active radiations (PAR), electron transport rate (ETR), current fluorescence (Ft) and reduced quantum yield (Y) in EMS line. Nitrogen treatments had no significant difference for panicle length, however, among genotypes, UAF-Q7 showed highest length of panicle followed by others. Among yield attributes, NIs-enriched urea expressed maximum 1000-seed weight and seed yield per plant in JQH-1 hybrid and EMS-line. Likely, an increase in quinoa grain protein contents was observed in JQH-1 hybrid for NIs-enriched urea. In conclusion, NIs-enriched urea with urease and nitrification inhibitors simultaneously can be used to improve the N uptake, seed yield and grain protein contents in quinoa, however, better crop response was attributed to enhanced plant growth and photosynthetic efficiency.

## 1. Introduction

Quinoa has exceptional nutritional grain value containing high protein contents and balanced amino acids while its enduring potential for abiotic stress tolerance makes it future potential crop both for nutritional and food security [1,2,3]. Since last decade, quinoa cultivation has spread into non-native geographical areas of world due to wide diversity of its ecotypes especially photoperiod response adaptation to specific agro-climatic conditions [1,2]. Nonetheless, quinoa growth and development are affected by environmental and genetic variations. For example, among environmental factors, nutrients especially nitrogen (N) improve vegetative growth in quinoa by affecting crop leaf area and growth rate, photosynthesis and N metabolism enzymes thereby increasing grain weight and yield [4,5,6]. These growth and yield responses in quinoa are variable to N supply and genotypic specific. The nitrogen use efficiency (NUE) is tightly linked to N uptake and its utilization efficiency (NUtE) that varies among genotypes, application method and time including rate in quinoa [6,7,8,9,10]. For instance, high total plant N uptake, its apparent recovery efficiency and harvest index including biomass and seed N contents was observed for 200 kg N ha^−1^, however, N utilization efficiency (NUtE) and remobilization from vegetative tissues to seed was found low in quinoa [9]. Improved growth and seed yield has also been reported in response to N application [10]. Likely, delayed flowering, extended seed filling period and improved photosynthetic pigments including seed yield was observed for 150 kg N ha^−1^ applied in two splits at 6–8 leaves and anthesis stages, respectively, compared to three splits and control with no N [11]. Nonetheless, variable response to N fertilization for growth including relative and crop growth rates, and seed yield was observed at low N rate in two quinoa cultivars [7]. Bascuñán-Godoy et al. [6] compared three quinoa cultivars similar in phenology for NUE traits under low and high N conditions. Photosynthetic rate, protein contents and leaf dry mass correlated positively with seed yield while proline contents, NH_4_^+^ assimilation and glutamine synthetase activity were correlated negatively under both N regimes. Nonetheless, high yields were correlated positively with seed weight under low N condition. Total N uptake in quinoa also vary with duration of crop growth cycle. Quinoa genotypes with shorter (NL-6) and longer growth duration (2-Want) accumulated similar total N before anthesis while differed after anthesis. Genotype 2-Want of longer growth duration accumulated 250 kg N ha^−1^ compared to NL-6 genotype with shorter growth behavior had reduced total uptake of 164 kg N ha^−1^ [8,12]. This variable response to N uptake after anthesis was associated with its remobilization towards reproductive structures [8].

Quinoa take up N as nitrates and application of N fertilizers had been found to improve its yield, water and NUE [13,14]. Among different sources, urea is mostly commonly used N source, most of which volatilizes as NH_3_, lost as NO_2_ into atmosphere and leaches as NO_3_ into the soil with low NUE which rarely exceeds 33% [15].

Among several strategies, application of urea enriched with urease and nitrification inhibitors can reduce N losses and improve crop productivity, hence NUE. Urease inhibitors reduce urea hydrolysis into NH_3_ by slowing down the urease enzyme activity [16,17]. This inhibited activity of urease decreases pH around urea molecules and NH_3_ concentration in soil, thus reducing its volatilization and increases the retention of applied N in soil to improve its plant availability for longer period [18]. On other hand, enhanced retention of NH_4_ ions into soil for longer time induces nitrification and dentification losses associated with NO_3_ leaching and N_2_O emission [19]. These leaching and denitrification losses can be minimized by nitrification inhibitors which limit oxidation of NH_4_^+^ to NO_3_^−^ by reducing the activity of nitrifying bacteria [20,21], however, retention of NH_4_^+^ by application of these inhibitors may further increase risk of volatilization [22,23]. Likely, boric acid has the potential to inhibit urease activity in soil [24]. While ammonium thiosulphate (ATS) as a good source of N and sulphur (S) for plants has the potential to inhibit both hydrolysis and nitrification without harming the soil microbial pool [25]. Therefore, rather than individual application, effective approach is to utilize these inhibitors in combination. The combined application of NIs reduces multiple losses associated with volatilization and denitrification [26,27] had been found to improve yield 5.7 and 8.0% of N uptake in rice [28], 22–36% increase in biomass and 23–32% of N uptake in pasture [29]. The combined application of different inhibitors, for instance, boric acid and 3, 4-Dimethylpyrazole phosphate (DMPP), urease and nitrification have also the potential to inhibit the N transformation synergistically [24], increase yield by 7.5% and NUE by 12.9% especially at low N dose [30,31], respectively. Nonetheless, these increases in N uptake and yield were associated with reduced losses of N and environmental footprints [24,28,29,30,31].

As uptake of N by quinoa varies with crop stage, response may be genotype specific and N application level, and no information is available for application of urea enriched with urease and nitrification inhibitors on growth, plant N uptake, photosynthetic efficiency, and yield response. Genotypes used in present study have contrasting behavior for growth including a cultivated variety, a hybrid and EMS line. Present study hypothesized that urea enriched with N inhibitors (NIs; boric acid, thiourea and sodium thiosulphate) improves its availability to quinoa plant at reduced N dose, thereby affecting crop performance and NUE.

## 2. Results

### 2.1. Plant Growth and Photosynthetic Pigments

Urea enriched with nitrogen inhibitors (35 kg N ha^−1^ + NI’s) improved the growth, chlorophyll values and plant N uptake in quinoa genotypes compared to control (0 kg N ha^−1^) and ordinary urea (70 kg N ha^−1^) (Table 1). There was an increase in shoot, root fresh and their dry weights, shoot and root lengths of quinoa genotypes UAF-Q7 and JHQ1 compared to control. Nonetheless, these increases in shoot dry weight, root fresh and dry weights were significantly similar to ordinary urea. Maximum and similar SPAD-chlorophyll values was found for enriched and ordinary urea application compared to control, while among genotypes, highest and significantly similar chlorophyll values were found between EMS-line and UAF-Q7 (Table 1).

### 2.2. Plant N Uptake and Nitrate Reductase Activity (NR)

**Figure 1:**
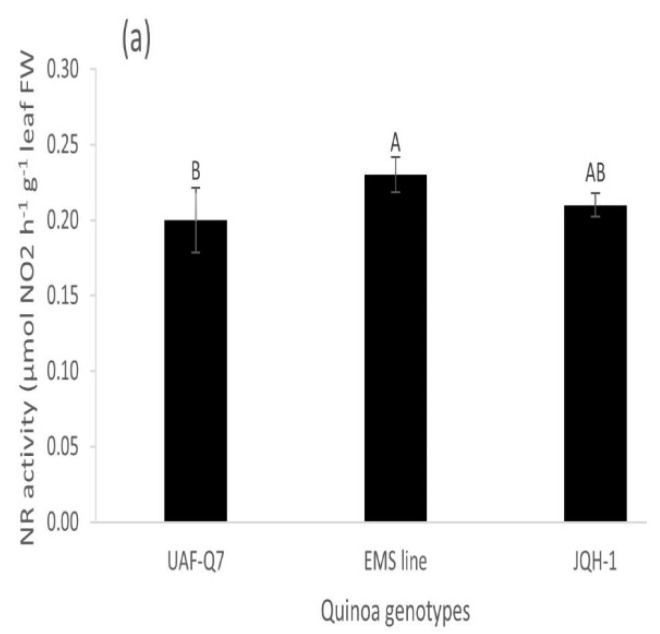

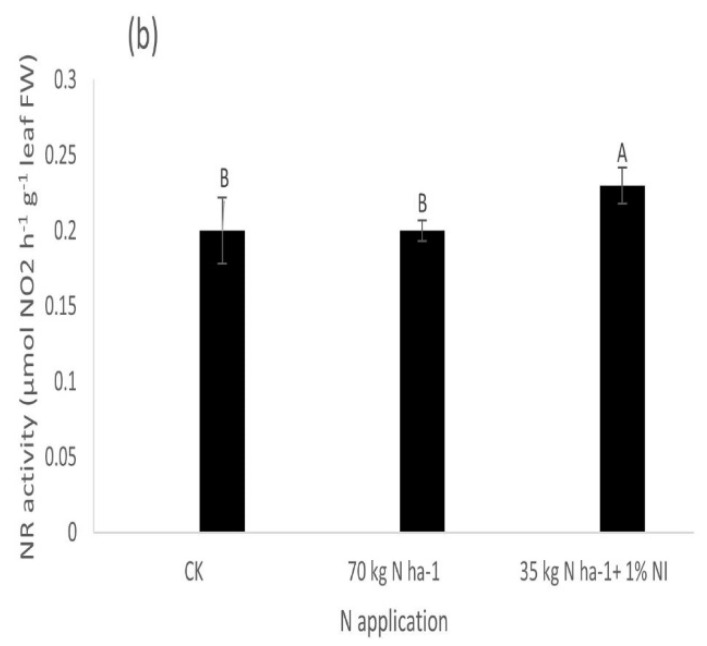
Nitrate reductase activities in three quinoa genotypes (**a**) and three fertilizer treatments (**b**) at bud formation stage Columns show mean of three replicates, whereas bar shows standard error. Means sharing same alphabets are not significantly different at *p* ≤ 0.5.

Application of NIs-enriched urea significantly improved the plant N uptake and NR activity in quinoa genotypes compared to control and vice versa response was observed for nitrogen utilization efficiency (NUtE). Urea enriched with NI’s showed highest plant N uptake in quinoa genotypes UAF-Q7 and EMS-line compared to control with minimum uptake. NIs-enriched urea application also expressed highest nitrate reductase (NR) activity in quinoa plants as compared to control and ordinary urea showing similar NR activity (Figure 1b). Among genotypes, EMS-line exhibited highest NR activity that was significantly similar to JQH-1 hybrid (Figure 1a).

### 2.3. Crop Phenology

Quinoa plants applied with NIs-enriched urea completed true and multiple leaf stage, bud formation, flowering, and maturity stages earlier than ordinary urea and control treatments. However, quinoa plants applied with enriched and ordinary urea attained panicle emergence, flowering and maturity at similar time compared to control with delayed in these attributes. Among genotypes, UAF-Q7 observed delayed bud formation and panicle emergence compared to other genotypes while all quinoa genotypes exhibited true and multiple leaf stage, flowering, and maturity at same time (Table 2).

### 2.4. Photochemical Efficiency Traits and SPAD-Chlorophyll Values

The photochemical efficiency traits and chlorophyll were affected significantly by application of NIs-enriched urea in quinoa genotypes. Quinoa plants applied with NIs-enriched urea showed highest photosynthetic active radiations (PAR), electron transport rate (ETR), current fluorescence (Ft) and reduced quantum yield (Y) in EMS line followed by two other genotypes. Lowes values for these attributes were observed in ordi nary urea compared to control. The Ft values and SPAD-chlorophyll values were similar when ordinary and NIs-enriched urea applied in UAF-Q7 and JQH-1 genotypes, respectively (Table 3).

### 2.5. Seed Yield and Its Attributes

Application of NIs-enriched urea produced tallest plants in genotype UAF-Q7 that was significantly similar to ordinary urea in same genotype. Minimum plant height was found in control plants with no supplemental N. There was no difference observed for panicle length in N treatments compared to control, however, among genotypes, UAF-Q7 expressed maximum panicle length followed by JQH1 hybrid.

Application of NIs-enriched urea showed maximum 1000-seed weight in quinoa genotypes UAF-Q7 and EMS-line. However, this increase was similar to ordinary urea in JQH-1 and EMS-line. Likely, highest seed yield per plant was found for NIs-enriched urea compared to ordinary urea while minimum seed yield per plant in control plants without supplemental N. Among genotypes, highest seed yield was expressed by JQH-1 hybrid followed by EMS-line while minimum in UAF-Q7 genotype.

### 2.6. Seed Protein Contents

Application of NIs-enriched urea showed highest seed protein contents in harvested grains of JHQ-1 hybrid that was similar to ordinary urea in same genotype. However, minimum seed protein contents were found in UAF-Q7 genotype in control treatment (Table 4).

## 3. Discussion

Urea fertilizers are often used as nitrogen (N) source worldwide due to high N contents. However, it is rapidly hydrolyzed to ammonia (NH_3_) and carbon dioxide (CO_2_) in soils. Nonetheless, application of urea enriched with urease and nitrification inhibitors are well known to synchronize N supply with crop demand to increase N use efficiency [32,33]. The present study evaluated the potential of urea enriched with N inhibitors (NI’s; 1% boric acid, thiourea and sodium thiosulphate + 35 kg N ha^−1^) to improve crop growth, photochemical efficiency, N uptake, seed yield and protein contents in contrasting quinoa genotypes compared to ordinary urea (70 kg N ha^−1^) and no N as control (0 kg N ha^−1^). The NIs enriched urea improved the growth, chlorophyll values and N uptake in quinoa genotypes [Table 1] are associated with its increased soil availability and is also an important component of chlorophyll structure [34]. Urea enriched with N-(n-butyl) thiophosphoric triamide (NBPT) inhibitor had been reported to improve photosynthetic pigments, plant growth and seed yield in cotton owing to increased N uptake. Likely, positive relationship of leaf N with chlorophyll contents has also been reported [34,35]. Increased N uptake and dry matter with application of urea enriched with NBPT in cotton [36] also reflected in plant fresh and dry biomass of quinoa when NIs-enriched urea was applied in this study [Table 1]. Increased NR activity in quinoa of present study [Table 1] was associated with beneficial effects of thiourea in enhancing N metabolism [36]. Thiourea application is reported to improve chlorophyll contents, photosynthetic activity, starch, and soluble protein levels in plants [37] confirms diverse functions of SH group in thiourea molecule. However, variable response of quinoa genotypes to NIs-enriched urea for total N uptake seems effect of NI’s to inhibit urease and nitrification [38]. Curti et al. [8] and Gomez et al. [12] reported that quinoa genotypes had no difference in total N content before anthesis independent of their growth duration which is also evident in present study where JHQ-1 hybrid showed low N uptake [Table 1] because of dilution effect associated with higher crop growth rate of quinoa hybrid not matching with N uptake [8,12]. Usually, application of N delays the crop phenological development, however, application of NIs-enriched urea helped quinoa plants to complete different stages of crop development earlier than ordinary urea and control treatments [Table 2]. There are no evidence showing the effects of NIs-enriched urea with NI’s on crop maturity, nevertheless, delay in development stages for N applied in splits had been reported in quinoa validated in present study findings [11]. Plant height and panicle length are genetic and stable characters, however, strongly affected by environmental factors including N. No significant difference observed between these traits for enriched and ordinary urea could be attributed to similar gains in photosynthetic efficiency of these genotypes [Table 4]. Nonetheless, increase in 1000-seed weight and seed yield of quinoa plant could be attributed to total N uptake which consistently contributed to enhance photosynthetic carbon fixation by affecting sink capacity [36,39]. The combined use of urease and nitrification inhibitors in NIs-enriched urea increased plant N uptake improving its efficiency associated with reduced losses, thereby increased yield and seed proteins contents [40]. The increase in seed protein contents in quinoa of present study by application of NIs-enriched urea [Table 4] were associated with slow-release effect to enhance retention and bioavailability of NH_4_-N for direct uptake and its remobilization to affect protein contents in grains [41,42]. Increase in protein contents with application of NH_4_-N [43] and inhibitors was associated with improved plant N uptake and its remobilization had also been reported in wheat and some other crops [44,45,46]. Gupta et al. [47] suggested that plants remobilize and constitute mechanism of re-uptake of resources such as NH_4_^+^ under N limited condition to ameliorate the increased NH_4_^+^ levels derived from different physiological process after anthesis. Nonetheless, present study results are in consistent with Bascuñán-Godoy et al. [48] where positive relationship between seed N% and seed amino acid was reported under low N supply and support the hypothesis to remobilize resources under limited vs. sufficient conditions [49].

## 4. Materials and Methods

### 4.1. Experimental Details

The seed of three quinoa genotypes, EMS-line and were collected from alternative crops lab, Department of Agronomy, University of Agriculture Faisalabad, Pakistan. These three genotypes varied in growth behavior and include a cultivated variety (UAF Q-7), a hybrid (JQH-1) and EMS line. The experiment was comprised of two factors as quinoa genotypes and fertilizer treatments including no N (0 kg N ha^−1^) (control), 75 kg N ha^−1^ (recommended) [50] and 35 kg N ha^−1^ enriched with 1% NI (Thiourea + Boric acid + Sodium thiosulphate). Experimental design used was completely randomized with two factors factorial. The tenth seed of each genotype were sown in earthen pots each filled with 5 kg soil at field capacity level under the wire house condition with exposure to natural growing condition. After seedling establishment, three plants per experimental unit were maintained for further growth and assays. The experimental treatments were randomized completely with factorial arrangement in three replications. Other fertilizers including phosphorus and potassium were thoroughly mixed in soil before sowing using 50 kg ha^−1^ of single super phosphate and 120 kg ha^−1^ of sulphate of potash, respectively. While half of nitrogen (N) was applied at sowing and other half at flowering stage. The pots were irrigated 10 and 15 days after sowing (DAS) for optimum growth of quinoa crop and later when required.

### 4.2. Determination of Plant Growth, Nitrogen Uptake, Utilization Efficiency and Seed Protein Contents

After 30–40 days of sowing at bud formation stage, only one plant was randomly uprooted for measuring plant growth traits including shoot and root lengths with measuring scale. The quinoa plant shoots fresh and root fresh weights was determined and oven-dried at 98 °C for 48 h for dry weights. The SPAD chlorophyll value of upper most leaf of quinoa plants was measured at the growth stage of BBCH 18 with the help of SPAD-501, Minolta Japan. For the determination of seedling N and grain protein, 0.5 g of plant and grain sample separately was taken into the Pyrex tube and added with 10 mL of concentrated H_2_SO_4_ for digestion and allowed to stand for overnight. After which, distillation was performed by Kjeldahl apparatus and then titrated against 0.1% H_2_SO_4_ [51]. Flasks were placed on the hot plate and heated at the temperature ranging between 100–150 °C for 30 min, then 2 mL of 30% H_2_O_2_ was added upon cooling, and heated again at increasing temperature of up to 300 °C. The processes were repeated till the solution became transparent and the final volume kept 50 mLby addition of distilled water. Distillation was performed by extracting 10 mLof the digested sample and 10 mLof 40% NaOH solution was added in the tube. The 5 mLboric acid solution (4%) was added to the receiver flask with 2–3 drops of mixed indicator and 40 mLof total solution was obtained by distillation and titrated against 0.01 N H_2_SO_4_ until the original color of methyl red appeared and the values were noted. For protein contents, nitrogen values were multiplied with a factor 5.95. Plant N uptake (mg N plant^−1^) and its utilization efficiency (NUtE; g DW per mg N) were measured as suggested by Merigout et al. [52] and Wang et al. [53], respectively.

### 4.3. Measurement of Nitrate Reductase Activity

At bud formation stage, leaf samples harvested at bud formation stage were shifted to icebox and stored at −30 °C until use [54]. For enzyme assay, leaf harvest (0.25 g) after grinding was extracted with 1 mL digestion buffer (1 mM EDTA + 10 mM cysteine) using ice-cold pestle mortar. The leaf extract was filtered through cheese cloth and homogenates was transferred to 2 mL falcon tubes, centrifuged at 10,000 rpm for 10 min to collect supernatant. Enzyme extract (1 mL) was added with 0.25 mL phosphate buffer (pH 7.5), 0.1 mL KNO_3_, 2 mM NADH and 0.35 mL distilled water to initiate reaction. After incubation at 30 °C for 15 min, reaction was terminated by the addition of 1% sulphanilamide and 0.02% naphthyl ethylenediamine reagent of 0.5 mL of each and kept it for 30 min to settle down. The absorbance of extract was measured on 540 nm using spectrophotometer (UV 4000, ORI Germany). The NR activity was calculated following Kaiser and Lewis [55].

### 4.4. Determination of Phenological Traits

All the crop growth stage of quinoa were recorded at regular intervals according to the BBCH scale [56]. The first true leaves (BBCH stage 11) developed 14–15 days after sowing and multiple leaf stage (BBCH 18) was recorded 25–26 days after sowing. The bud formation stage after 35–40 days of sowing was coded as BBCH 50. The panicle emergence (BBCH 59) started after the 50–55 days of sowing and flowering (BBCH 67) in the inflorescence of quinoa plant after the 70–75 days after sowing. After flowering, the grains of quinoa start to ripened and milky stage recorded after 90–95 after sowing was coded as BBCH 81 and the maturity stage after 120–130 days after sowing as BBCH 90.

### 4.5. Measurement of Photosynthetic Efficiency and SPAD-Chlorophyll Value

Photochemical efficiency traits were measured at 12:00–1:00 pm in a bright sunny-day using photosynthetic efficiency analyzer (MINI-PAM-II) and chlorophyll values by SPAD-chlorophyll meter (SPAD-501, Minolta, Osaka, Japan) at panicle emergence stage (BBCH 59–60) from the upper most leaf. The data regarding current fluorescence value (Ft), electron transport rate (ETR), photosynthetically active radiations (PAR) and effective photochemical yield (Fv/Fm) was measured.

### 4.6. Seed Yield and Its Related Traits

At physiological maturity, height of plant and main panicle length was measured with a measuring scale. At harvesting, grains number per plant and 1000-grain yield were recorded. The grains number per plant was counted by threshing the panicles and 1000 grain weight was measured by using seed counter.

### 4.7. Statistical Analysis

Analysis of variance technique was performed to analyze the data statistically using Statistix 8.1 software (Hamburg, Germany) and differences among treatment means were computed by least significance difference (LSD) test at 5% probability.

## 5. Conclusions

The present study showed that enriching urea with urease and nitrification inhibitors (1% boric acid, thiourea and sodium thiosulphate) simultaneously have potential to reduce crop N requirements and its application can improve the N use efficiency, seed yield and grain protein contents in quinoa irrespective of genotypes. Nonetheless, better crop response at (NI; 1% boric acid, thiourea and sodium thiosulphate + 35 kg N ha^−1^) was attributed to photosynthetic efficiency and increased N uptake in quinoa.

## Figures and Tables

**Table 1:** NIs-enriched urea effects on growth, plant N uptake, nitrogen utilization efficiency (NUtE) and SPAD-chlorophyll in three quinoa genotypes at bud formation stage.

**Quinoa Genotypes**	**Shoot Fresh Weight (g)**				**Root Fresh Weight (g)**			
	**CK (0 kg N ha^−1^)**	**70 kg N ha^−1^**	**35 kg N ha^−1^ + 1% NI**	**Means Genotypes**	**CK (0 kg N ha^−1^)**	**70 kg N ha^−1^**	**35 kg N ha^−1^ + 1% NI**	**Means Genotypes**
UAF-Q7	6.10 d	12.35 b	18.10 a	12.18 A	0.30 bc	0.40 ab	0.53 a	0.41 A
EMS line	9.27 c	4.73 d	11.50 bc	8.50 B	0.33 bc	0.10 d	0.40 ab	0.28 B
JQH-1	4.20 d	12.77 b	16.20 a	11.06 A	0.17 cd	0.33 bc	0.33 bc	0.28 B
Means N	6.52 A	9.95 B	15.27 A		0.27 B	0.28 B	0.42 A	
HSD	G = 1.36, N = 1.36, G × N = 2.36				G = 0.10, N = 0.10, G × N = 0.18			
**Quinoa Genotypes**	**Shoot Dry Weight (g)**				**Root Dry Weight (g)**			
	**CK (0 kg N ha^−1^)**	**70 kg N ha^−1^**	**35 kg N ha^−1^ + 1% NI**	**Means Genotypes**	**CK (0 kg N ha^−1^)**	**70 kg N ha^−1^**	**35 kg N ha^−1^ + 1%NI**	**Means Genotypes**
UAF-Q7	0.82 cde	1.17 abc	1.48 a	1.15	0.06 b	0.08 ab	0.11 a	0.08 A
EMS line	0.10 bcd	0.49 e	1.24 ab	0.90	0.06 b	0.02 c	0.07 b	0.05 B
JQH-1	0.60 de	1.33 ab	1.51 a	1.15	0.02 b	0.06 b	0.08 ab	0.06 B
Means	0.80 B	0.10 B	1.41 A		0.05 B	0.05 B	0.09 A	
HSD	G = n.s., N = 0.24, G × N = 0.42				G = 0.02, N = 0.02, G × N = 0.03			
**Quinoa Genotypes**	**Shoot Length (cm)**				**Root Length (cm)**			
	**CK (0 kg N ha^−1^)**	**70 kg N ha^−1^**	**35 kg N ha^−1^ + 1% NI**	**Means Genotypes**	**CK (0 kg N ha^−1^)**	**70 kg N ha^−1^**	**35 kg N ha^−1^ + 1% NI**	**Means Genotypes**
UAF-Q7	16.00 e	23.80 b	24.15 b	21.31 B	5.50 e	6.80 de	11.50 a	7.93
EMS line	21.77 bc	6.67 de	23.50 b	20.64 B	8.30 c	7.05 cd	7.40 cd	7.58
JQH-1	19.60 cd	28.00 a	29.00 a	25.53 A	9.80 b	7.85 cd	8.10 cd	8.58
Means	19.12 c	22.8 B	25.55 A		7.88 B	7.23 B	9.00 A	
HSD	G = 1.81, N = 1.80, G × N = 3.12				G = n.s., N = 0.86, G ×N = 1.49			
**Quinoa Genotypes**	**SPAD-Chlorophyll Values**				**Plant N Uptake (mg N Plant^−1^)**			
	**CK (0 kg N ha^−1^)**	**70 kg N ha^−1^**	**35 kg N ha^−1^ + 1% NI**	**Means Genotypes**	**CK (0 kg N ha^−1^)**	**70 kg N ha^−1^**	**35 kg N ha^−1^ +1%NI**	**Means Genotypes**
UAF-Q7	40.60	44.40	45.10	43.37 AB	1.71 d	3.31 c	6.84 a	3.95
EMS line	46.00	44.55	45.73	45.43 A	3.00 c	1.67 d	4.99 b	3.22
JQH-1	35.90	44.03	43.47	41.13 B	1.12 d	3.58 c	5.92 ab	3.53
Means	40.83 B	44.33 A	44.77 A		1.94 C	2.82 B	5.92 A	
HSD	G = 2.96, N = 2.96, G × N = n.s.				G = n.s., N = 0.66, G × N = 1.15			
**Quinoa Genotypes**	**NUtE (g DW per mg N)**							
	**CK (0 kg N ha^−1^)**	**70 kg N ha^−1^**	**35 kg N ha^−1^ + 1% NI**	**Means Genotypes**				
UAF-Q7	0.51 a	0.38 bc	0.26 e	0.42 A				
EMS line	0.35 c	0.31 d	0.26 e	0.36 B				
JQH-1	0.39 b	0.39 bc	0.27 de	0.26 C				
Means	0.39 A	0.31 C	0.35 B					
HSD	G = 0.02, N = 0.02, G × N = 0.04							

Letters among and within columns denote significant differences in means for nitrogen and between cultivars at *p* ≤ 0.05.

**Table 2:** Influence of NIs-enriched urea on phenological development in three quinoa genotypes.

**Quinoa Genotypes**	**Days to True Leaf**				**Days to Multiple Leaf**			
	**CK (0 kg N ha^−1^)**	**70 kg N ha^−1^**	**35 kg N ha^−1^ + 1% NI**	**Means Genotypes**	**CK (0 kg N ha^−1^)**	**70 kg N ha^−1^**	**35 kg N ha^−1^ + 1% NI**	**Means Genotypes**
UAF-Q7	19.67 a	13.33 c	14.00 c	15.67	28.33 ab	26.33 cde	25.33e	26.67
EMS line	18.33 ab	19.00 a	15.67 bc	17.67	29.33 a	28.00 abc	26.00 de	27.78
JQH-1	18.33 ab	18.00 ab	13.33 c	16.56	26.67 bcde	27.00 bcde	27.67 abcd	27.11
Means N	18.78 A	16.78 B	14.33 C		28.11 A	27.11 AB	26.33 C	
HSD	G = n.s., N = 1.59, G × N = 2.75				G = n.s., N = 1.08, G × N = 1.87			
**Quinoa Genotypes**	**Days to Bud Formation**				**Days to Panicle Emergence**			
	**CK (0 kg N ha^−1^)**	**70 kg N ha^−1^**	**35 kg N ha^−1^ + 1% NI**	**Means Genotypes**	**CK (0 kg N ha^−1^)**	**70 kg N ha^−1^**	**35 kg N ha^−1^ + 1%NI**	**Means Genotypes**
UAF-Q7	40.33	40.00	40.33	40.22 A	59.67	59.00	56.67	58.44 A
EMS line	40.00	39.67	36.67	38.78 B	55.67	57.33	55.33	55.44 B
JQH-1	37.33	38.67	36.33	37.44 C	59.00	54.67	53.33	55.66 B
Means	39.22 A	39.44 A	37.78 B		58.11 A	55.44 B	55.67 B	
HSD	G = 1.15, N = 1.15, G × N = n.s.				G = 1.82, N = 1.82, G ×N = n.s.			
**Quinoa Genotypes**	**Days to Flowering**				**Days to Maturity**			
	**CK (0 kg N ha^−1^)**	**70 kg N ha^−1^**	**35 kg N ha^−1^ + 1% NI**	**Means Genotypes**	**CK (0 kg N ha^−1^)**	**70 kg N ha^−1^**	**35 kg N ha^−1^ + 1% NI**	**Means Genotypes**
UAF-Q7	79.33	77.67	77.00	78.00	127.67	125.33	121.67	124.89
EMS line	79.33	78.00	76.00	77.78	128.00	124.00	122.67	124.89
JQH-1	78.00	77.00	75.33	76.78	125.33	126.00	122.67	124.67
Means	78.88 A	77.56 AB	76.11 B		127.00 A	125.11 AB	122.33 B	
HSD	G = n.s., N = 1.97, G × N = n.s.				G = n.s., N = 3.55, G × N = n.s.			

Letters among and within columns denote significant differences in means for nitrogen and between cultivars at *p* ≤ 0.05.

**Table 3:** Effects of NI-enriched urea on photochemical efficiency and SPAD-chlorophyll values in three quinoa genotypes at panicle emergence stage.

Quinoa Genotypes	SPAD-Chlorophyll				Photosynthetic Active Radiation (PAR)			
	CK (0 kg N ha^−1^)	70 kg N ha^−1^	35 kg N ha^−1^ + 1% NI	Means Genotypes	CK (0 kg N ha^−1^)	70 kg N ha^−1^	35 kg N ha^−1^ + 1% NI	Means Genotypes
UAF-Q7	43.77 bc	46.93 ab	48.07 a	46.26 A	887.00 d	584.70 ef	1268.70 b	913.44 A
EMS line	42.33 c	34.90 d	48.30 a	41.84 B	486.00 f	584.70 ef	1445.70 a	838.78 B
JQH-1	35.65 d	47.97 a	47.80 ab	43.81 B	657.00 e	649.70 e	1074.30 c	793.67 B
Means N	40.58 C	43.27 B	48.06 A		676.70 B	606.30 C	1262.90 A	
HSD	G = 2.42, N = 2.42, G × N = 4.19				G = 67.743, N = 67.743, G × N = 117.33			
**Quinoa Genotypes**	**Electron Transport Rate (ETR)**				**Current Fluorescence Value (Ft)**			
	**CK (0 kg N ha^−1^)**	**70 kg N ha^−1^**	**35 kg N ha^−1^ + 1% NI**	**Means Genotypes**	**CK (0 kg N ha^−1^)**	**70 kg N ha^−1^**	**35 kg N ha^−1^ + 1%NI**	**Means Genotypes**
UAF-Q7	214.07 b	169.07 bc	176.97 bc	186.70 AB	471.67 bc	607.33 a	450.00 bc	509.67
EMS line	164.47 bc	139.83	294.43 a	199.58 A	422.00 c	423.33 c	635.00 a	493.44
JQH-1	126.87 c	148.23 c	184.13 bc	153.08 B	467.00 bc	386.00 c	556.33 ab	469.78
Means	168.47 B	152.38 B	218.51 A		453.56 B	472.22 B	547.11 A	
HSD	G = 36.703, N = 36.703, G × N = 63.57				G = n.s., N = 71.709, G × N = 124.20			
**Quinoa Genotypes**	**Quantum Yield (Y)**							
	**CK (0 kg N ha^−1^)**	**70 kg N ha^−1^**	**35 kg N ha^−1^ + 1% NI**	**Means Genotypes**				
UAF-Q7	0.62 a	0.57 ab	0.42 c	0.54				
EMS line	0.57 ab	0.63 a	0.50 bc	0.57				
JQH-1	0.47 c	0.60 a	0.44 c	0.51				
Means	0.55 A	0.6 A	0.46 B					
HSD	G = n.s., N = 0.0470, G × N = 0.0814							

Letters among and within columns denote significant differences for nitrogen and between cultivars at *p* ≤ 0.05.

**Table 4:** Effects of NI-enriched urea on yield components and seed protein contents in three quinoa genotypes at maturity.

**Quinoa Genotypes**	**Plant Height (cm)**				**Panicle Length (cm)**			
	**CK (0 kg N ha^−1^)**	**70 kg N ha^−1^**	**35 kg N ha^−1^ + 1% NI**	**Means Genotypes**	**CK (0 kg N ha^−1^)**	**70 kg N ha^−1^**	**35 kg N ha^−1^ + 1% NI**	**Means Genotypes**
UAF-Q7	70 b	81 a	88 a	80 A	27	23	26	25 A
EMS line	41 c	49 c	43 c	44 B	15	15	13	14 C
JQH-1	51 c	49 c	49 c	50 B	18	19	20	19 B
Means N	54	60	60		20	19	19	
HSD	G = 5.61, N = n.s., G × N = 9.72				G = 2.59, N = n.s., G × N = n.s.			
**Quinoa Genotypes**	**1000 Seed Yield (g)**				**Seed Yield Per Plant (g)**			
	**CK (0 kg N ha^−1^)**	**70 kg N ha^−1^**	**35 kg N ha^−1^ + 1% NI**	**Means Genotypes**	**CK (0 kg N ha^−1^)**	**70 kg N ha^−1^**	**35 kg N ha^−1^ + 1%NI**	**Means Genotypes**
UAF-Q7	0.28 d	0.44 bc	0.53 a	0.42 B	1.16	2.32	2.92	2.13 C
EMS line	0.42 c	0.47 abc	0.54 a	0.48 A	2.46	3.17	3.93	3.19 B
JQH-1	0.48 abc	0.52 ab	0.52 ab	0.51 A	3.40	3.54	5.33	4.09 A
Means	0.39 C	0.48 B	0.53 A		2.34 C	3.01 B	4.06 A	
HSD	G = 0.05, N = 0.05, G × N = 0.08				G = 0.60, N = 0.59, G × N = n.s.			
**Quinoa Genotypes**	**Seed Protein Contents (%)**							
	**CK (0 kg N ha^−1^)**	**70 kg N ha^−1^**	**35 kg N ha^−1^ + 1% NI**	**Means Genotypes**				
UAF-Q7	10.16 d	13.22 c	15.66 b	13.01 B				
EMS line	14.66 bc	14.83 bc	15.66 bc	15.05 A				
JQH-1	14.32 bc	16.44 ab	18.8 a	16.53 A				
Means	13.05 A	14.83 B	16.7 A					
HSD	G = 1.5758, N = 1.5758, G × N = 2.72							

Letters among and within columns denote significant differences in means for nitrogen and between cultivars at *p* ≤ 0.05.

## Data Availability

Data is contained within the article.
